# Evaluation of Antibacterial and Anticancer Characteristics of Silver Nanoparticles Synthesized from Plant Extracts of *Wrightia tinctoria* and *Acacia chundra*

**DOI:** 10.1155/2023/6352503

**Published:** 2023-03-20

**Authors:** Anitha Jegadeeshwari, Narasimha Reddy Seelam, Venkata Ratnam Myneni

**Affiliations:** ^1^Department of Chemical Engineering, Rajalakshmi Engineering College, Chennai-602105, India; ^2^Department of Chemical Engineering, Mettu University, Metu, Ethiopia

## Abstract

The study showed the ability to synthesize environmentally friendly silver nanoparticles (AgNPs) using extracts from *Wrightia tinctoria* seeds and *Acacia chundra* stems. Surface plasmon resonance peaks in the UV-Vis absorption spectra of both plant extracts verified AgNP synthesis. The structural and morphological properties of the AgNPs were investigated using analytical techniques such as XRD, FTIR, TEM, and EDAX. The AgNPs have an FCC crystalline structure, according to XRD study, and their sizes range from 20 to 40 nm, according to TEM images. Based on the results, these plant extracts have been identified as suitable bioresources for AgNP production. The study also showed that both AgNPs had significant levels of antibacterial activity when tested on four different microbial strains using the agar-well diffusion method. The bacteria tested included two Gram-positive strains (*Staphylococcus aureus* and *Micrococcus luteus*) and two Gram-negative strains (*Proteus vulgaris* and *Escherichia coli*). Furthermore, the AgNPs were found to have a significant anticancer effect on MCF-7 cell lines, suggesting that they may be useful in therapeutic applications. Overall, this research highlights the potential of the plant extracts considered as a source for synthesizing eco-friendly AgNPs with potential applications in medicine and other fields.

## 1. Introduction

Nanotechnology enables the production of materials with a wide range of morphologies, dimensions, and chemical compositions, all of which fall within the range of 1–100 nanometers. Nanoparticles have a wide range of uses in electronics, biology, and medicine due to their optical, antibacterial, therapeutic, and catalytic capabilities [1, 2]. The higher the ratio of surface area to volume of the nanoparticles is, the more efficient they are in biological systems. Traditional methods of chemical and physical synthesis of nanomaterials require more time and involve the use of more hazardous reducing or stabilizing chemicals [1–3]. Plants contain bioactive components such as phenols, flavonoids, saponins, glycosides, tannins, alkaloids, polysaccharides, proteins, terpenoids, amines, ketones, and aldehydes that act as reducing, stabilizing, and capping agents in the conversion of metal ions to metal nanoparticles. Hence, the use of plant resources will eliminate the need for toxic solvents and chemicals [1–8]. The aforementioned issues can be addressed, and biocompatibility with nanoparticles can be obtained, by using plant extracts as reducers and stabilizers in the synthesis of ecologically friendly metallic nanoparticles [9, 10].

Pharmaceuticals, coatings, biological labeling, and packaging are just few of the industries where nanoparticles might be useful [5–9]. Silver nanoparticles (AgNPs) have attracted a lot of attention from researchers. In spite of the fact that higher concentrations of silver are toxic, a number of studies [8–10] demonstrate that lower concentrations of AgNO_3_ have superior chemical stability and catalytic activity, biocompatibility, and intrinsic therapeutic potential. Researchers from every corner of the globe are interested in silver nanoparticles because of the remarkable antibacterial qualities that they possess. Tribal plants are revered in India as the ecosphere's equivalent of a botanical garden because they are used to treat a wide range of illnesses and physical distress and are also used as medicines. The bulk of the country's rural population practices traditional Indian medicine, such as Ayurveda [11]. The employment of nanoparticles in conjunction with well-known and extensively distributed traditional plants has thrown fresh light on the study of natural science. There have been a lot of studies conducted on how to make silver nanoparticles with potential anticancer and antibacterial action. Some examples include *Arctium lappa* fruit extract [3], *Solanum melongena* leaves [3], *Taraxacum mongolicum* leaves [3], red currants berries [4], alcoholic flower extracts [5], *Pisum sativum* L extracts [6], *Mangifera indica* seed extracts [7], berry extracts [9], *Cucumis prophetarum* leaf extracts [10], and *Alpina katsumadai* seed extract.

The *Wrightia tinctoria* (Wt) tree is an *Apocynaceae* family member that is found in several places in India [12, 13]. It is a deciduous tree of modest to medium size. The seeds and the bark of this plant are both employed in the practice of traditional Indian medicine as a means of curing gastrointestinal conditions including diarrhea and dysentery. This plant's bark has been demonstrated to be useful in treating a range of diseases, including abdominal pain, skin infections, and wounds; it can even reverse the effects of snake venom [14]. Furthermore, the seeds of this plant have been shown to have aphrodisiac properties [12–15]. *Wrightia tinctoria* is mentioned in several traditional medicinal systems used in South Asia as a useful cure for the treatment of heart palpitations and excessive blood pressure. Previous studies on the *Wrightia tinctoria* plant discovered important chemical components such as alkaloids, triterpenoids, steroids, and flavonoids [14]. *Acacia catechu* (Ac), commonly known as Katha or Karangali, is a well-known medicinal shrub in India [14–17]. Catechins, flavanol glycosides, flavonal dimers, caffeine, and rhamnetin are some of the plant's helpful components [18]. The antibacterial and wound-healing abilities of this plant have been studied. It is used alone or in combination with cinnamon or opium to treat chronic diarrhea. *Acacia catechu* water decoctions are commonly drunk as health beverages, particularly in the Indian state of Kerala and neighboring states in India's southern region [18, 19]. The water decoction is supposed to enhance skin complexion, cleanse the blood of pollutants, and boost the immune system. The anti-inflammatory qualities of stem bark extract are one of its pharmacological capabilities. When tested against the MCF-7 cell line, the methanolic extract of *Acacia catechu* stem bark was found to be effective as both an antioxidant and an anticancer agent [20]. This discovery was made possible because methanolic extraction is more stable than water extraction.

**Figure 1:**
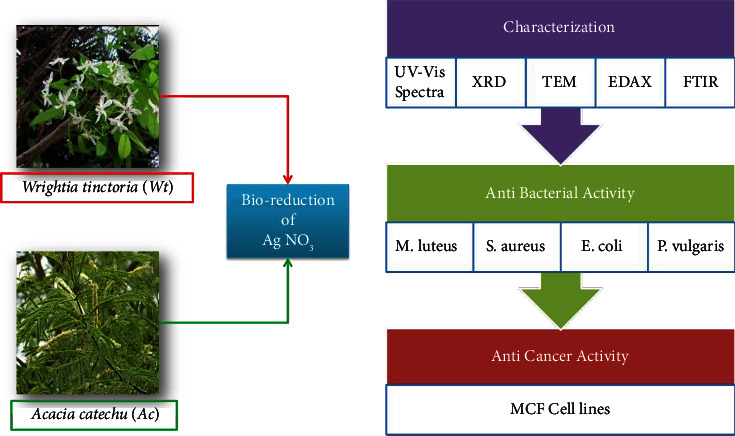
Schematic representation of the present study.

Our goal in this study is to develop an easy green synthesis of AgNPs using *Wrightia tinctoria* seeds and *Acacia chundra* stems. The generated AgNPs were analyzed by UV-Vis spectroscopy, X-ray diffraction (XRD), Fourier-transform infrared spectroscopy (FTIR), transmission electron microscopy (TEM), and energy dispersive X-ray spectroscopy (EDAX). All of the AgNPs' characteristics, including their crystalline nature, size, and surface features, were investigated. An investigation of the biological activities of biosynthesized AgNPs was conducted. We used the well diffusion experiment to test the antibacterial activity of the AgNPs against both Gram-positive (*Micrococcus luteus* and *Staphylococcus aureus*) and Gram-negative bacteria (*Escherichia coli* and *Proteus vulgaris*). Furthermore, the anticancer potential of the produced AgNPs has been investigated.  Figure 1 presents the schematic representation of the experimental process.

## 2. Materials and Methods

All the reagents in this study were of analytical grade, used without further purification. The *Wrightia tinctoria* (Wt) seeds were obtained in the SP. Kovil, Kancheepuram, and *Acacia catechu* (Ac) stem bark were collected from Chennai district.

### 2.1. Preparation of Extract Using Seeds of *Wrightia tinctoria* (Wt)

The seeds of the Wrightia tinctoria plant were thorough washings, first with water from the running faucet, then with distilled water twice, to remove any dirt or other organic particles that may have contaminated them. They are left to air-dry in the shade for a period of 20 days before being ground into a powder using a standard blender. The powder is kept in a container that is hermetically sealed and placed in the refrigerator. A 250 ml conical flask was filled with 10 g of powder and 200 ml of distilled water. The solution was brought to a temperature of 60°C and then boiled for two hrs. After being allowed to cool, the extract solution was passed through a Whatman 41 filter to remove any particles that were still present. In the end, the Wt extract solution was retained for use after being stored in a refrigerator at a temperature of 40°C.

### 2.2. Preparation of Extract Using Stem of *Acacia catechu* (Ac)

Following a comprehensive cleaning, the stems of *Acacia catechu* were allowed to dry at room temperature. The stem was cut into very small pieces, and then it was boiled for 20 min in a round-bottom flask containing 250 ml of distilled water. For the purpose of filtering the extract, Whatman No. 1 filter paper was utilized. The produced extract was placed in storage for use in further studies.

### 2.3. Synthesis of AgNPs

To convert the Ag^+^ ions, 25 ml of the plant extract sample is combined with 100 ml of an aqueous solution that contains 1 nM of AgNO_3_ [21]. After 24 hrs, the reaction mixture was settled to its final state. Silver nanoparticle production was inferred from the change in reaction mixture colour. To further separate the reaction mixture, it was centrifuged at 2,000 rpm for 10 min. After that, the AgNPs were separated from the sample by centrifuging it for 15 min at a speed of 16,000 rpm. The AgNPs were collected and dispersed in acetone before being stored.

### 2.4. AgNP Characterization

The identification of silver nanoparticles was confirmed, and their physio-chemical properties were studied through several characterization methods. The AgNP formation was confirmed by using a Shimadzu UV-1800 spectrophotometer. The spectral data were collected in the 200–800 nm range. The X-ray diffraction measurements were taken with a Bruker diffractometer outfitted with Cu radiation (=1.5406). For FTIR study, a Perkin Elmer spectrometer was used, and spectra were gathered in the 500–4000 cm^−1^ region. The TEM study was performed using the JEOL JEM-2100 microscope. The nanoparticle solution was dried on a copper matrix to prepare the samples for analysis.

### 2.5. Antibacterial Activity

In this research, four bacterial strains were chosen: two Gram-positive (*Micrococcus luteus* and *Staphylococcus aureus*) and two Gram-negative (*Proteus vulgaris* and *Escherichia coli*) to assess the antibacterial effectiveness of the synthesized AgNPs using the Agar-well diffusion method. These bacterium isolates were acquired from the National Chemical Laboratory in Pune, India, and were kept at 4°C until further use. These microorganisms are clinically important because they can induce a variety of diseases. The AgNPs were synthesized in a 100% DMSO solution at four distinct concentrations (25, 50, 75, and 100 *μ*g/ml). Each well received 100 *μ*l of an enriched AgNP sample. To assess antibacterial effectiveness, the radius of the zone of inhibition around each well was determined. Three different experiments were performed, and the average of the data was used to determine AgNPs' antibacterial efficacy.

### 2.6. Anticancer Activity

Cancer is a hereditary illness that quickly disseminates throughout the body, causing irreparable damage to human tissue [18]. Breast cancer, which is the growth of malignant cells in the breast, is a significant cause of high female cancer mortality rates. In this work, the in vitro anticancer effects of SeWt-AgNPs and StAc-AgNPs were investigated on MCF-7 cell lines at varying dosages using the MTT test. The National Centre for Cell Science in Pune, India, provided the MCF-7 breast cancer cell line that was used in this study. Cells were planted at a density of 1 × 10^4^/well in 96-well plates and allowed to grow for 24 hrs at 37°C in a humidified atmosphere. After that, they were given an AgNP treatment at concentrations of 1.5, 6.25, 12.5, 50, and 100 *μ*g/ml and placed in an incubator for 24 hrs. The MTT test was used in accordance with the procedure to determine the level of viability shown by cells that had been treated with these compounds as well as those that had been left untreated (serving as a control). The expression was what was used to determine whether or not the cells were viable.

(1) $$\begin{array}{c} \%\;viability=\left[\frac{\left(mean\;absorbance\;of\;treated\;group\right)}{\left(mean\;absorbance\;of\;control\;group\right)}\right]\times 100\text{.} \end{array}$$

## 3. Results and Discussion

### 3.1. Characterization Studies of the Silver Nanoparticles

#### 3.1.1. UV-Vis Analysis

**Figure 2:**
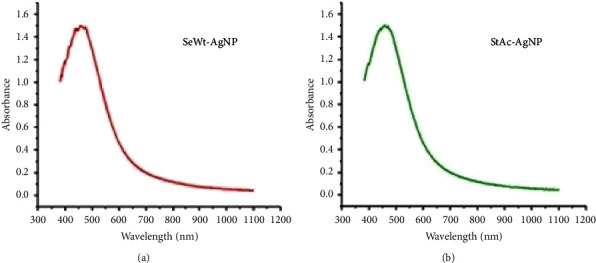
UV-visible spectroscopy graph for (a) AgNPs synthesized from seed extracts of *W. tinctoria* and (b) AgNPs synthesized from stem extracts of *A. catechu*.

The formation of AgNPs is typically indicated by a change in the colour of the reaction mixture, which changed from colourless to yellowish-brown after 24 hrs in this research, showing that the plant extracts used were successful at reducing silver nitrate. A UV-Vis spectrometer was used to examine the surface plasmon resonance (SPR) region during AgNP production. The amount of peaks in the SPR band, their occurrence, and their changes over time provide crucial information for describing the nanoparticles' properties. The UV-Vis spectra of SeWt-AgNPs and StAc-AgNPs revealed absorption peaks at 443 nm and 440 nm, respectively (as shown in Figures 2(a) and 2(b)). Furthermore, no typical peaks were detected, suggesting that the solution did not contain an overabundance of starting materials. These findings support earlier research by demonstrating the effective synthesis of AgNPs using plant extracts [21–24]. Rabaa Algotiml et al. [1] synthesized biogenic AgNPs from extracts of three distinct types of marine algae (green, brown, and red algae), and the absorption maxima were found to be at 424, 409, and 415 nm, respectively. Similarly, peaks at 420 nm were detected when the surface plasmon resonance (SPR) band of silver nanoparticles prepared using Calotropis procera latex was observed by a UV-Vis spectrophotometer [23]. Previous research and the current results show the possibility of using plant extracts to make eco-friendly AgNPs with a variety of absorption spectra that can be used in a variety of uses, including medicine and technology.

#### 3.1.2. XRD Studies

**Figure 3:**
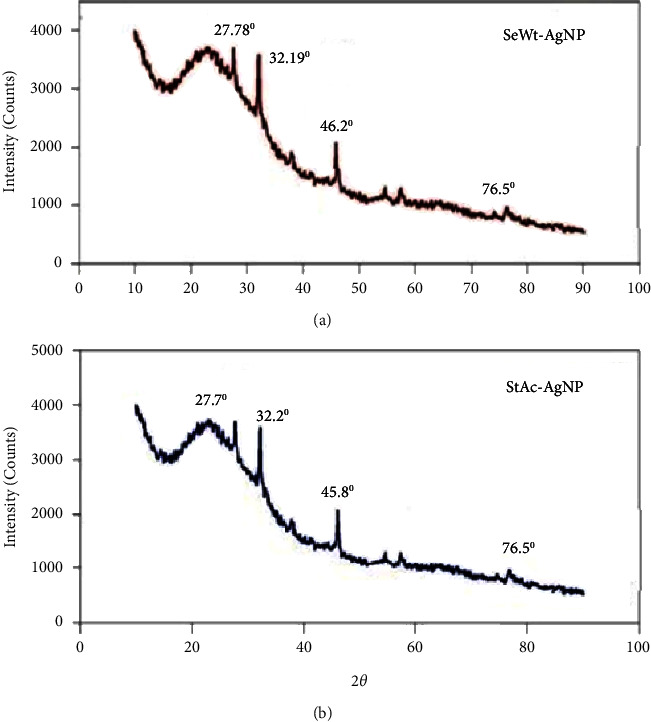
XRD peaks for (a) AgNPs synthesized from seed extracts of *W. tinctoria* and (b) AgNPs synthesized from stem extracts of *A. catechu*.

The X-ray diffraction (XRD) is widely used to study the structural and crystalline properties of materials. The structural properties of SeWt-AgNPs and StAc-AgNPs were investigated using XRD analysis in this study. The XRD patterns found for both samples verified the existence of a crystalline structure and matched the elemental silver face-centered cubic (FCC) lattice structure. The appearance of four different peaks in the XRD spectra of SeWt-AgNPs (Figure 3(a)) at 27.78°, 32.19°, 46.2°, and 76.5° and StAc-AgNPs at 27.7°, 32.2°, 45.8°, and 76.5° (Figure 3(b)) showed the FCC crystal structure of the synthesized AgNPs. Some minor peaks in the XRD spectra were also detected, which could be ascribed to surface reactions of either biological or chemical origin. The findings of a number of other researchers were similar to the conclusion reached in the present investigation [3–8].

#### 3.1.3. TEM Analysis

**Figure 4:**
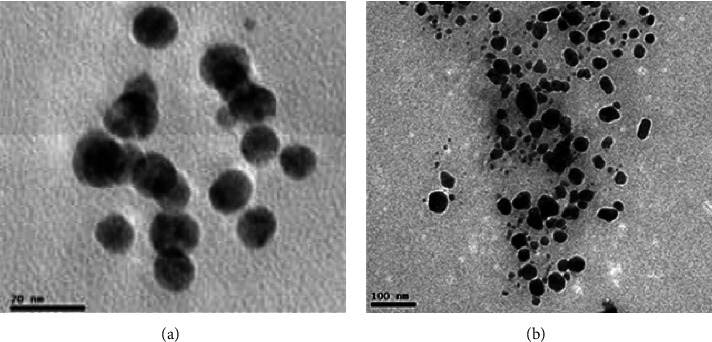
HR-TEM images for (a) AgNPs synthesized from seed extracts of *W. tinctoria* and (b) AgNPs synthesized from stem extracts of *A. catechu*.

**Figure 5:**
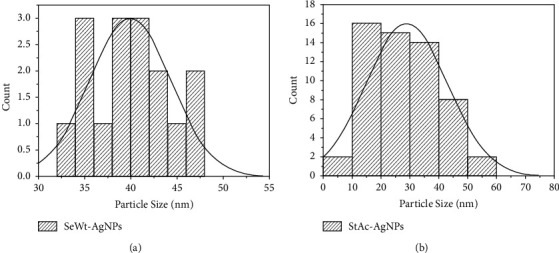
Particle size distributions for (a) AgNPs synthesized from seed extracts of *W. tinctoria* and (b) AgNPs synthesized from stem extracts of *A. catechu*.

Transmission electron microscopy (TEM) was utilized for the purpose of determining the morphology, shape, and size of nanoparticles. Figures 4(a) and 4(b) show the TEM micrograph of SeWt-AgNPs and StAc-AgNPs, respectively. It was discovered that the majority of the created AgNPs were in the shape of spheres. The TEM pictures were processed with the image J program, which resulted in the creation of a histogram displaying the distribution of particle sizes. It has been determined that the diameters of SeWt-AgNPs and StAc-AgNPs are between 20 and 40 nm, with the former having a value that is considerably greater than the latter. SeWt-AgNPs had a median particle size of 39.75 nm (Figure 5(a)) whereas the StAc-AgNPs had a median particle size of 27.79 nm (Figure 5(b)). In their study using seeds from the *Mangifera indica* plant, Donga and Chanda [7] found spherical AgNPs of varied sizes from 9 to 61 nm. The extract of *Alpinia katsumadai* seed produced quasispherical AgNPs with an average particle size of 12 nm [12].

#### 3.1.4. EDAX Spectra Analysis

**Figure 6:**
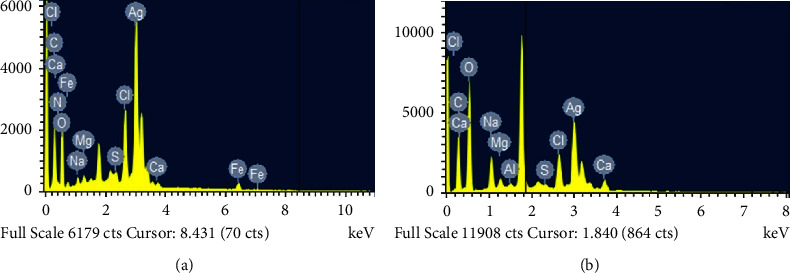
EDX results showing presence of silver element in (a) SeWt-AgNPs and (b) StAc-AgNPs.

Energy dispersive X-ray spectroscopy, often known as EDAX, is a method that may be utilized to ascertain the elemental components that are present in the specimen. It was determined that surface Plasmon resonance was the cause of the absorption peak that was observed in the EDAX spectra (Figures 6(a) and 6(b)), which was located at around 2.5 keV. This provides more evidence that silver ions can be broken down into elemental silver [25].

#### 3.1.5. FTIR Spectral Analysis

**Figure 7:**
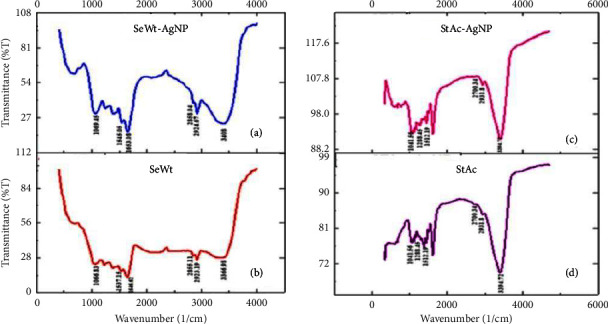
FTIR peaks showing (a) functional groups of SeWt-AgNPs, (b) functional groups of SeWt, (c) functional groups of StAc-AgNPs, and (d) functional groups of StAc.

The FTIR studies reveal the peaks in the produced SeWt-AgNPs at 1069 cm^−1^, 1545 cm^−1^, 1653 cm^−1^, 2858 cm^−1^, 2924 cm^−1^, and 3408 cm^−1^, respectively (Figure 7(a)). In the FTIR spectra of SeWt (Figure 7(b)), bands could be seen at 1066 cm^−1^, 1537 cm^−1^, 1646 cm^−1^, 2855 cm^−1^, 2923 cm^−1^, and 3366 cm^−1^. The peaks at 1066 cm^−1^ belong to the C–H bending vibration, the bands at 1537 cm^−1^ relate to the N–O stretching vibrations, 1646 cm^−1^ corresponds to the diketone stretching vibrations, and 3366 cm^−1^ refers to the stretching vibration of –OH groups. The shift in the peak values slightly, from 1066 cm^−1^ to 1069 cm^−1^ and from 1537 cm^−1^ to 1545 cm^−1^, in case of SeWt-AgNPs corresponds to the phytochemicals that were involved in the reduction and stabilization of AgNPs. The FTIR spectra of AgNPs (Figure 7(d)) revealed the existence of bands at the following wavelengths: 1041 cm^−1^, 1288 cm^−1^, 1512 cm^−1^, 2700 cm^−1^, 2931 cm^−1^, and 3394 cm^−1^. The peaks at 1066 cm^−1^ correspond to C–H bending vibrations, the bands at 1288 cm^−1^ correspond to –OH stretching vibrations, 2700 cm^−1^ and 2923 cm^−1^ correspond to asymmetric –CH2–, symmetric –CH3, and –CH2 stretching, and 3394 cm^−1^ corresponds to the stretching vibration of bonded and nonbonded O–H groups, with minor shifts after the formation of the StAc-AgNPs (Figure 7(c)).

### 3.2. Antibacterial Activity

**Figure 8:**
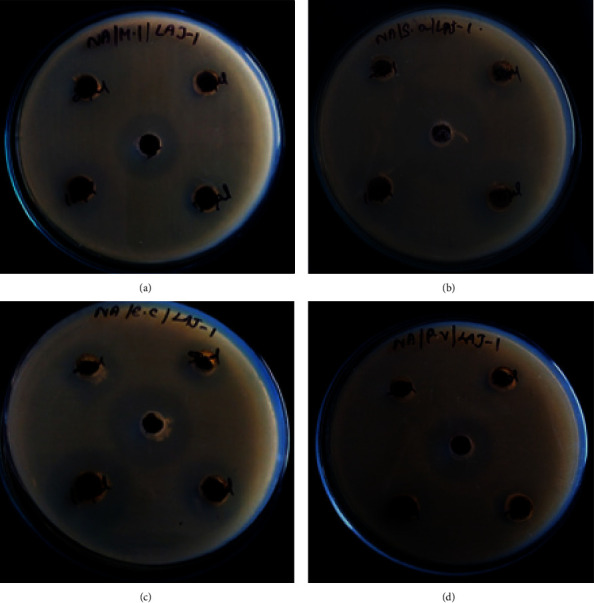
Antibacterial activity showing inhibition zone when treated by SeWt-AgNPs against (a) *M. luteus,* (b) *S. aureus,* (c) *E. coli*, and (d) *P. vulgaris.*

**Figure 9:**
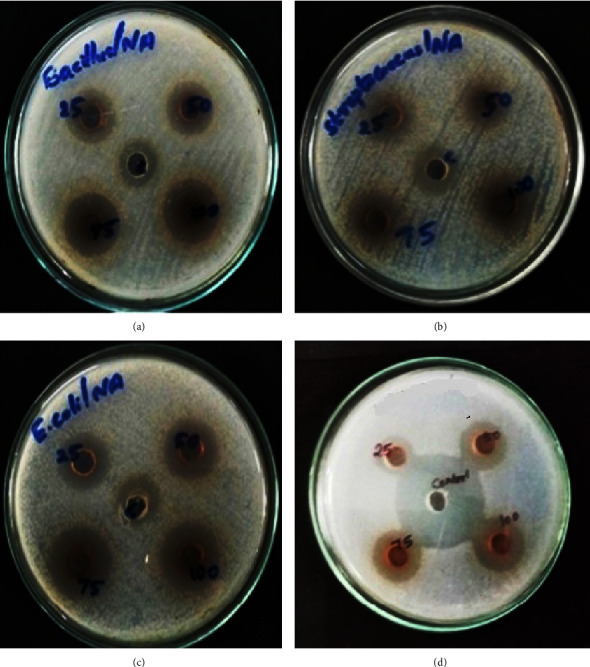
Antibacterial activity showing inhibition zone when treated by StAc-AgNPs against (a) *M. luteus,* (b) *S. aureus,* (c) *E. coli*, and (d) *P. vulgaris.*

Against a diverse collection of microorganisms, the antibacterial efficacy of the AgNPs was evaluated at four distinct concentrations: 25, 50, 75, and 100 *μ*g/ml (Figures 8 and 9). When compared to StAc-AgNPs, it has been demonstrated that SeWt-AgNPs possess antibacterial activity that is considerably less potent. At each concentration, the SeWt-AgNPs demonstrated their antibacterial activity. When Gram-negative bacteria were exposed to SeWt-AgNPs, they displayed an antibacterial activity that was remarkably similar to antibacterial activity of Gram-positive bacteria. When compared to their antibacterial activity against other bacteria, StAc-AgNPs have a level of effectiveness that is marginally higher against *E. coli* and *B. subtilis*. The antibacterial activity varies based on the concentration of the nanoparticles as well as the type of bacteria being studied. All of the sensitive bacteria exhibit growth-restriction zones with a diameter that ranges from 11 mm to 20 mm. Silver nanoparticles are among the most significant metal nanoparticles because of their robust antibacterial capabilities. Because of these features, silver nanoparticles are among the most significant types of metal nanoparticles. The AgNPs, which were produced from an aqueous extract of the outer peel of Pisum sativum, were shown to be bactericidal against four human pathogenic bacteria (with inhibition zones ranging from 8.70 to 11.10 mm on agar plates) [6]. It has been discovered that green silver nanoparticles (AgNPs) produced from an aqueous extract of *Mangifera indica* seeds are more efficient than traditional antibacterial treatments against Gram-negative bacteria [7].  Table 1 contains a discussion of the findings of microbial efficacy of AgNPs produced from different plant extracts.

### 3.3. Anticancer Activity

**Figure 10:**
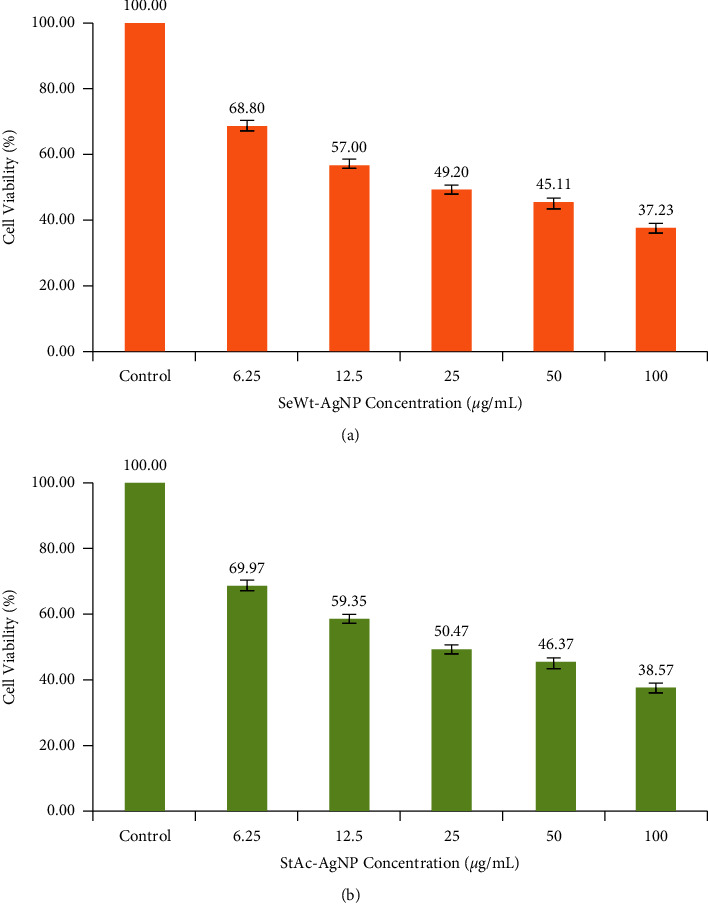
MCF-7 cell viability after treatment with varied AgNP concentrations of (a) SeWt-AgNPs and (b) StAC-AgNPs.

**Figure 11:**
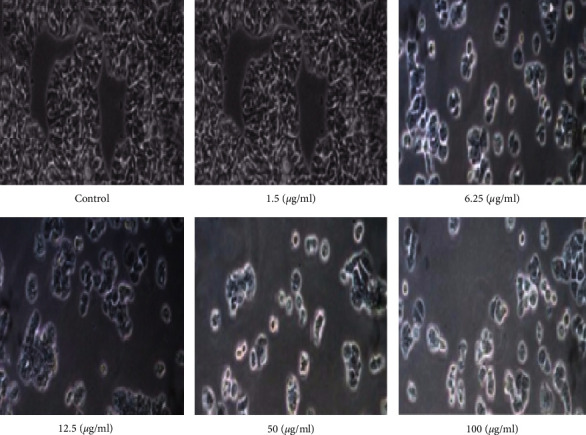
Morphological alterations in MCF-7 lines caused by different dosages of SeWt-AgNPs.

**Figure 12:**
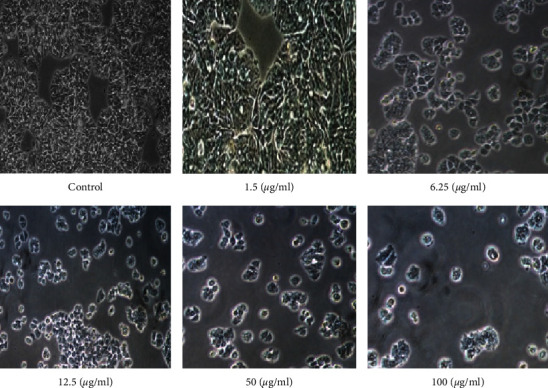
Morphological alterations in MCF-7 lines caused by different dosages of StAc-AgNPs.

AgNP cell viability was measured using the MTT test. When delivered at concentrations between 1.5 and 100 *μ*g/ml, both SeWt-AgNPs and StAc-AgNPs show significant cytotoxicity (Figure 10). It was shown that both types of AgNPs were able to prevent the formation of cancer cells by the fact that the proliferation of the MCF cell line was dramatically decreased across the board. Light microscopy was used to examine the morphology of MCF-7 cancer cells after they had been incubated with AgNPs for 24 hrs at doses of 1.5, 6.25, 12.5, 50, and 100 *μ*g/ml (Figures 11 and 12). At concentrations of 1.5 *μ*g/ml of AgNPs, there are no discernible alterations in the morphology of the photos. However, at 6.25, 12.5, 50, and 100 *μ*g/ml, AgNPs inhibited cell proliferation at a dose-dependent rate. There was a noticeable shift in cell shape at both 50 and 100 *μ*g/ml. The results demonstrated a significant reduction in MCF-7 cell viability as compared to the plant extract. We can attribute this decline to the presence of AgNPs.  Table 2 shows the findings of a study contrasting the antineoplastic activity of the various natural sources used to produce AgNPs.

## 4. Conclusions

The *Wrightia tinctoria* seed extracts and the *Acacia catechu* stem extracts were both utilized during the course of this experiment to facilitate the synthesis of the AgNPs. UV-Vis spectrophotometry confirms the formation of AgNPs, and the XRD analysis reveals the FCC crystalline structure of the AgNPs. The TEM micrographs demonstrated that the AgNPs had a spherical shape and that their diameters ranged from 20 to 40 nm. The AgNPs were found to be uniformly distributed. Overall, the study has shown that plant extracts can be used as eco-friendly and cost-effective alternatives to traditional chemical methods for the synthesis of AgNPs. The synthesized AgNPs showed promising antibacterial and anticancer activities, indicating their potential use in various biomedical applications. Further research is needed to explore the mechanisms of action and toxicity of these AgNPs and to optimize their synthesis parameters to enhance their properties for specific applications.

## Figures and Tables

**Table 1:** Comparative study of various natural sources used for the preparation of AgNPs and their antibacterial activity.

S. No.	Source	Antibacterial activity against	Application	Citation
1	*Tinospora cordifolia* (stem)	*P. aeruginosa*	Resistance to multidrug resistant bacteria	[26]
2	*Cucumis sativus*	*Mycobacterium tuberculosis*	Healing of wounds	[27]
3	*Calotropis procera* (leaves)	*Vibrio cholera*	Treatment of waterborne diseases	[28]
4	*Acalypha indica* (leaf)	*E. coli* and *Vibrio cholera*	Reduction of microbial loading in drinking water	[29]
5	*Taraxacum officinale* (leaf)	*P. syringae*	Disease management	[30]
6	*Boerhaavia diffusa*	*F. branchiophilum*	Protection of fishery livestock	[31]
7	*Wrightia tinctoria* (seeds)	*B. subtilis, S. aureus, E. coli,* and *P. vulgaris*	Inhibition of bacterial infection in target organism	Current study
8	*Acacia catechu* (stem)			

**Table 2:** Comparative study of various natural sources used for the preparation of AgNPs and their antineoplastic activity.

S. No.	Source	Anticancer activity against	Application	Citation
1	*Gymnema sylvestre*	HT29	Inhibition of growth of the human colon carcinoma	[32]
2	*Hibiscus sabdariffa*	U87	Inhibition of growth of the human brain carcinoma	[33]
3	*Moringa oleifera*	A549 and SNO	Inhibition of growth of the human lung and esophageal carcinoma	[34]
4	*Podophyllum hexandrum*	HeLa	Inhibition of growth of the human lung and cervical carcinoma	[35]
5	*Rhus chinensis*	MKN-28, Hep3B, and MG-63	Inhibition of growth of the human liver and stomach carcinoma	[36]
6	*Wrightia tinctoria* (seeds)	MCF-7	Inhibition of growth of the breast carcinoma	Current study
7	*Acacia catechu* (stem)			

## Data Availability

The data used in this study are included within the article.
